# Evolution of Structural and Magnetic Properties of Fe-Co Wire-like Nanochains Caused by Annealing Atmosphere

**DOI:** 10.3390/ma14164748

**Published:** 2021-08-23

**Authors:** Marcin Krajewski, Mateusz Tokarczyk, Sabina Lewińska, Katarzyna Brzózka, Kamil Bochenek, Anna Ślawska-Waniewska

**Affiliations:** 1 Institute of Fundamental Technological Research, Polish Academy of Sciences, Pawińskiego 5B, 02-106 Warsaw, Poland; kboch@ippt.pan.pl; 2 Faculty of Physics, University of Warsaw, Pasteura 5, 02-093 Warsaw, Poland; Mateusz.Tokarczyk@fuw.edu.pl; 3 Institute of Physics, Polish Academy of Sciences, Al. Lotników 32/46, 02-668 Warsaw, Poland; lewinska@ifpan.edu.pl (S.L.); slaws@ifpan.edu.pl (A.Ś.-W.); 4 Faculty of Mechanical Engineering, Department of Physics, University of Technology and Humanities, Stasieckiego 54, 26-600 Radom, Poland; k.brzozka@uthrad.pl

**Keywords:** annealing, amorphous materials, Fe-Co nanochains, magnetic-field-induced synthesis, Wire-like nanostructure

## Abstract

Thermal treatment is a post-synthesis treatment that aims to improve the crystallinity and interrelated physical properties of as-prepared materials. This process may also cause some unwanted changes in materials like their oxidation or contamination. In this work, we present the post-synthesis annealing treatments of the amorphous Fe_1−*x*_Co*_x_* (*x* = 0.25; 0.50; 0.75) Wire-like nanochains performed at 400 °C in two different atmospheres, i.e., a mixture of 80% nitrogen and 20% hydrogen and argon. These processes caused significantly different changes of structural and magnetic properties of the initially-formed Fe-Co nanostructures. All of them crystallized and their cores were composed of body-centered cubic Fe-Co phase, whereas their oxide shells comprised of a mixture of CoFe_2_O_4_ and Fe_3_O_4_ phases. However, the annealing carried out in hydrogen-containing atmosphere caused a decomposition of the initial oxide shell layer, whereas a similar process in argon led to its slight thickening. Moreover, it was found that the cores of thermally-treated Fe_0.25_Co_0.75_ nanochains contained the hexagonal closest packed (hcp) Co phase and were covered by the nanosheet-like shell layer in the case of annealing performed in argon. Considering the evolution of magnetic properties induced by structural changes, it was observed that the coercivities of annealed Fe-Co nanochains increased in comparison with their non-annealed counterparts. The saturation magnetization (*M*_S_) of the Fe_0.25_Co_0.75_ nanomaterial annealed in both atmospheres was higher than that for the non-annealed sample. In turn, the *M*_S_ of the Fe_0.75_Co_0.25_ and Fe_0.50_Co_0.50_ nanochains annealed in argon were lower than those recorded for non-annealed samples due to their partial oxidation during thermal processing.

## 1. Introduction

Among many well-known preparation processes of nanomaterials, precipitation or co-precipitation is one of the most frequently used synthesis methods in modern nanotechnology [1,2,3]. This approach offers the production of a high quantity of material during one run of the synthesis and at the same time is relatively easy-to-perform. The precipitation synthesis is associated with the chemical reduction of liquid or semi-liquid precursors (gels) using both inorganic and organic reducing agents [4,5,6,7]. Among them, sodium borohydride (NaBH_4_) is a commonly chosen agent to produce various inorganic nanoparticles due to its strength, availability, as well as safety [8,9,10].

The usage of NaBH_4_ as the reducing agent in precipitation method usually leads to the formation of nanoparticles [3,9,10,11,12,13]. However, it has been recently demonstrated that the presence of an external magnetic field during the synthesis of iron and/or cobalt nanoparticles can cause their alignment and results in the formation of iron, cobalt or iron–cobalt Wire-like nanostructures [14,15,16,17]. Their morphologies and structural properties are very specific because they look like long chains of nanoparticles.

It is also known that the nanomaterials synthesized with NaBH_4_ often reveal amorphous or nanocrystalline nature mainly due to the incorporation of a very small amount of boron [18]. This causes the post-synthesis thermal treatment to be required in order to improve their crystallinity as well as resulting physical properties. In general, this process is not difficult to perform in the laboratory or industrial scale but it should be kept in mind that the nanostructures composed of magnetically important elements like Fe, Ni, and Co are extremely sensitive for oxidation at elevated temperatures even in the atmospheres containing low content of oxygen [9,19,20,21]. In order to limit the possibility of oxide formation, their thermal treatments can be carried out in the inert gases, for instance, argon and nitrogen [22,23,24,25,26]. Considering this approach, the main purpose of this work was to investigate the thermal treatment of the amorphous Fe-Co Wire-like nanochains with various iron-to-cobalt ratios synthetized in the magnetic-field-induced (MFI) co-precipitation process [16,17] in two different atmospheres. One of them was a dangerous mixture of 80% nitrogen and 20% hydrogen, whereas the second one was cheaper and safer argon atmosphere. After that, we found that these two post-synthesis processes caused significantly different changes of structural and magnetic properties of the investigated Fe-Co nanostructures. The obtained results are collected and comprehensively described in this work. Moreover, it is worth noting that they can be considered as an important source of information for the application of the thermally-treated iron-cobalt Wire-like nanochains in the magnetic and microwave absorbers [27,28] or catalysis [29,30].

## 2. Experimental

### 2.1. Fabrication of Iron-Cobalt Wire-like Nanostructures

The Fe_1−*x*_Co*_x_* (*x* = 0.25; 0.50; 0.75) Wire-like nanochains were synthetized according to our previous reports describing the MFI co-reduction reaction [16,17]. In typical synthesis, the proper amounts of iron(II) chloride hydrate (FeCl_2_·*x*H_2_O; 98%, Carl Roth GmbH, Karlsruhe, Germany) and cobalt(II) chloride hexahydrate (CoCl_2_·6H_2_O; 97%, Carl Roth GmbH) were dissolved in 300 mL of deionized water. These mixtures served as the reaction precursors. In turn, 1.4 g of sodium borohydride (NaBH_4_; 97%, Carl Roth GmbH) dissolved in 175 mL of deionized water was used as a reducing agent and was dropped to the previously prepared precursor solutions. The process was carried out in the inert argon gas (>99%, BialGaz Company, Białystok, Poland) and also in the average external magnetic field of about 0.05 T induced by two parallel neodymium magnets.

The as-prepared nanomaterials were rinsed three times with ethanol (99.8%, Avantor (POCH), Gliwice, Poland) and then three times with acetone (99.5%, Carl Roth GmbH) in order to clean the as-prepared Fe-Co nanochains. After that, they were dried at 50 °C in a vacuum for 2 h.

The as-prepared Fe_0.75_Co_0.25_, Fe_0.50_Co_0.50_, and Fe_0.25_Co_0.75_ nanochains were subjected to a thermal treatment in a tubular furnace in two different atmospheres. One of them was a mixture of 80% nitrogen and 20% hydrogen (>99%, Multax S.C. Company, Zielonki-Parcela, Poland), whereas the second one was argon (>99%, BialGaz Company). In general, the samples were heated from room temperature to 400 °C with heating rate of 10 °C per minute. Then, they were held at this temperature for 0.5 h. After that, they were gently cooled down to room temperature. At this point, it should also be mentioned that the Fe-Co nanochains were heated at 400 °C in order to omit their sintering. Moreover, the thermally-treated samples in hydrogen-containing atmosphere were assigned as Fe_0.75_Co_0.25_ H_2_, Fe_0.50_Co_0.50_ H_2_, and Fe_0.25_Co_0.75_ H_2_ whereas the annealed chains in argon atmosphere were assigned as Fe_0.75_Co_0.25_ Ar, Fe_0.50_Co_0.50_ Ar, and Fe_0.25_Co_0.75_ Ar.

### 2.2. Characterization of Wire-like Nanostructures

The morphologies and structural properties of as-prepared and thermally treated Fe-Co nanochains were determined with a Zeiss Crossbeam 350 scanning electron microscope (SEM, Oberkochen, Germany) equipped with an EDAX Elite Plus energy dispersive X-ray spectrometer (EDS, Berwyn, Pennsylvania, USA), a JEOL—JEM 1011 transmission electron microscope (TEM, Tokyo, Japan), a Philips X’Pert Diffractometer (XRD, Malvern, United Kingdom) equipped with a Cu X-ray lamp, and a POLON transmission Mössbauer spectrometer (TMS, Kraków, Poland).

The SEM and TEM measurements were carried out at 5 and 80 kV of accelerating voltage, respectively. Each Fe-Co sample for the SEM investigations was glued to a carbon-type covering an alumina holder. In parallel to SEM imaging, the chemical compositions of as-prepared Fe-Co samples were determined using a EDS technique. The areal EDS data was collected mostly from the nanochains. However, some unwanted signal coming from the conductive carbon-type was also recorded during the EDS measurements mainly due to a thin layer of glued samples. In order to collect the TEM images, the sample was suspended in acetone (99.8%, Avantor (POCH)), sonicated for 2 min, and then dropped onto a nickel TEM grid coated with holey carbon films (Electron Microscopy Sciences) and air-dried. At this point, it should be underlined that all samples were prepared according to the same above-mentioned procedures.

The X-ray diffraction data was collected using a Cu X-ray lamp (λ = 1.5406 Å). The Fe-Co powders were placed on a single crystalline silicon holder. The patterns were collected at room temperature in scattering angle (2θ) range of 30–90° with a step of 0.05° and a scan rate of 0.2 °/min.

The TMS measurements were performed at room temperature using a ^57^Co(Rh) gamma radiation source and a conventional spectrometer operating in vertical geometry. The source of γ-radiation was additionally placed on a vibrator head moving longitudinally to the gamma beam with a speed varying linearly with time. The collected Mössbauer spectra were analyzed using the specialized NORMOS program (POLON, Kraków, Poland) applying a minimization procedure and the method of least squares with additional constraints. Relative intensity of the sub-spectra was estimated on the basis of the surface area under the line of the given component. The determined isomer shift values were referred to the signal of pure iron measured at room temperature.

The magnetic properties were determined at room temperature recording the magnetic hysteresis loops of the as-prepared and thermally-treated Fe-Co materials with a Quantum Design Physical Property Measurement System equipped with a vibrating sample magnetometer (VSM) option. At this point, it should be added that the saturation magnetization (M_S_) values were determined by fitting to the high-field part of the measured hysteresis loops to the following Equation (1):

M(B) = M_S_·(1 − a/B − b/B^2^) (1)

where B is the applied magnetic field, and a and b are constants.

## 3. Results

### 3.1. Electron Microscopy Investigations

**Figure 1:**
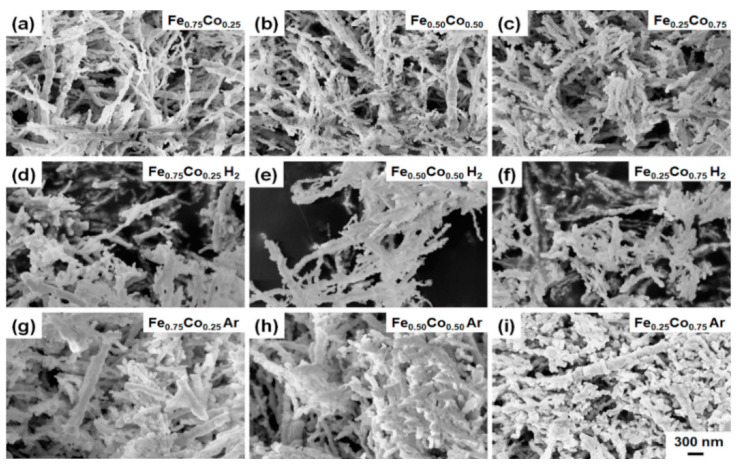
SEM images of as-prepared (**a**) Fe_0.75_Co_0.25_, (**b**) Fe_0.50_Co_0.50_, and (**c**) Fe_0.25_Co_0.75_ Wire-like nanochains; SEM images of (**d**) Fe_0.75_Co_0.25_, (**e**) Fe_0.50_Co_0.50_, and (**f**) Fe_0.25_Co_0.75_ Wire-like nanochains annealed in hydrogen-containing atmosphere; SEM images of (**g**) Fe_0.75_Co_0.25_, (**h**) Fe_0.50_Co_0.50_, and (**i**) Fe_0.25_Co_0.75_ Wire-like nanochains annealed in argon.

The morphology and the internal structure of as-prepared as well as annealed Fe-Co nanostructures were characterized using SEM and TEM, respectively. Figure 1 presents the SEM images. Analyzing them, one can see that the as-prepared samples are composed of nanoparticles aligned in the nearly straight chains. The diameters of nanoparticles forming chain-like nanostructures are not uniformly distributed even between single neighboring particles. Their values contain between 60 to 180 nm for all as-prepared samples but the average diameters of the Fe_0.75_Co_0.25_, Fe_0.50_Co_0.50_, and Fe_0.25_Co_0.75_ particles equal 102, 99, and 108 nm, respectively. Considering the lengths of as-prepared chains, they can reach even over 3 µm but their average value is around 1.2 µm. Analyzing the SEM results, it is also found that the annealing of the Fe-Co nanochains in hydrogen-containing atmosphere does not influence their size. In turn, their thermal treatment in argon leads to an increase of their average diameters of about 20 nm.

**Figure 2:**
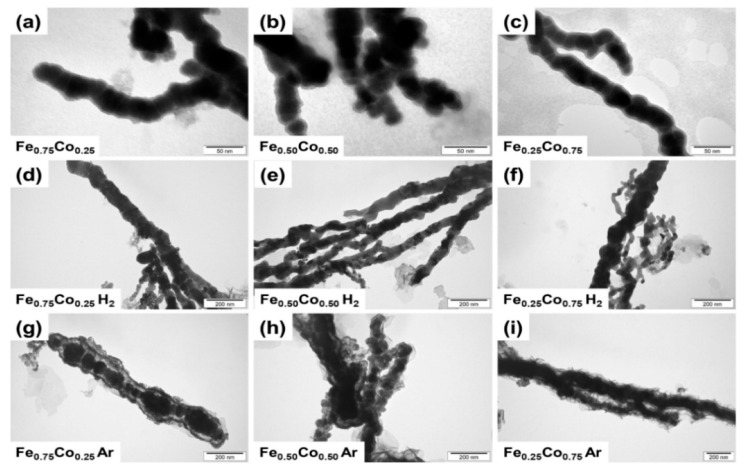
TEM images of as-prepared (**a**) Fe_0.75_Co_0.25_, (**b**) Fe_0.50_Co_0.50_, and (**c**) Fe_0.25_Co_0.75_ Wire-like nanochains; TEM images of (**d**) Fe_0.75_Co_0.25_, (**e**) Fe_0.50_Co_0.50_, and (**f**) Fe_0.25_Co_0.75_ Wire-like nanochains annealed in hydrogen-containing atmosphere; TEM images of (**g**) Fe_0.75_Co_0.25_, (**h**) Fe_0.50_Co_0.50_, and (**i**) Fe_0.25_Co_0.75_ Wire-like nanochains annealed in argon.

Since the SEM measurements do not allow us to analyze the internal changes caused by annealing, the TEM studies were performed and their results are shown in Figure 2. The images obtained for as-prepared Fe-Co nanochains confirm the SEM observations that they are composed of nanoparticles aligned in nearly straight chains. However, these samples also reveal characteristic core-shell structure since a dark core region and a thin light-grey shell layer are clearly visible in the TEM images (cf. Figure 2a–c). The thickness of shell layer is quite uniform for all as-prepared samples and does not exceed 4 nm. The annealing process of the Fe-Co nanochains in argon causes thickening of the shell layer. It should be also noted that the Fe_0.75_Co_0.25_ and Fe_0.50_Co_0.50_ samples are covered by a well adherent layer on which some small nanoparticles are formed. In the case of the Fe_0.25_Co_0.75_ nanochains, their shell layer takes the form of nanosheets, which roll up their cores. In turn, the thermal treatment in hydrogen-containing atmosphere seems to cause a decomposition of the initial shell layer because no light-grey shell color related to its present is visible in the TEM images.

### 3.2. Determination of Chemical Composition

The chemical composition of the as-prepared Fe-Co nanochains was determined with the EDS measurements, which were performed in parallel to their SEM imaging. The obtained results are collected in Table 1. Analyzing the presented data, one can see that the elemental contribution associated with boron and oxygen in the samples may be disturbed by the carbon tape. However, considering the actual share between Fe and Co atoms in the as-prepared Fe-Co nanochains corresponds well to the composition of initial ion precursors taken to the MFI synthesis. The obtained EDS results also show that the as-prepared samples are slightly contaminated by the compounds containing boron and sodium, whereas no presence of chlorine atoms was detected. Besides that, they might be partly oxidized due to the presence of oxygen whose content is higher than the previously recalled boron, sodium, and carbon.

### 3.3. XRD Investigations

**Figure 3:**
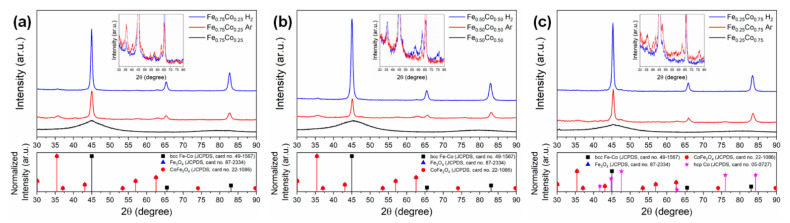
XRD patterns of as-prepared and annealed (**a**) Fe_0.75_Co_0.25_, (**b**) Fe_0.50_Co_0.50_, and (**c**) Fe_0.25_Co_0.75_ Wire-like nanochains; the bottom panels indicate the reference positions of the Bragg peaks based on the JCPDS database for the crystalline bcc Fe-Co, Fe_3_O_4_, CoFe_2_O_4_, and hcp Co phases; the insets present the magnified view of XRD patterns associated with oxides in the range between 30 and 80°.

The evolution of the structural properties between non-annealed and annealed Fe-Co nanochains was traced using the XRD technique. The collected XRD patterns are shown in Figure 3. Analyzing them, one can see that all as-prepared nanochains are nanocrystalline or amorphous because only two very broad peaks are present in the XRD patterns. Their 2θ positions cannot be easily assigned to the well-defined crystallographic planes of body-centered cubic (bcc) Fe-Co phase (JCPDS card no. 49-1567). It is also important that no reflexes associated with oxides are found for the non-annealed samples. This indicates that they are amorphous. In turn, it is evident that the thermal treatment in the argon or in hydrogen-containing atmosphere leads to the significant improvement of crystallographic ordering in the Fe-Co nanochains. All XRD patterns recorded for the annealed samples reveal the well-oriented peaks of bcc Fe-Co phase. Besides that, the weak signal coming from a mixture of Fe_3_O_4_ (JCPDS card no. 87-2334) and CoFe_2_O_4_ (JCPDS card no. 22-1086) appears in the diffractograms collected for the Fe_0.75_Co_0.25_ and Fe_0.50_Co_0.50_ samples annealed in both argon and hydrogen-containing atmosphere. In fact, it is hard to differentiate which oxide dominates in the investigated nanochains because their 2θ positions are very similar. It should be also mentioned that the thermally-treated Fe_0.25_Co_0.75_ sample contains hexagonal closest packed (hcp) Co phase (JCPDS card no. 05-0727).

### 3.4. Mössbauer Spectroscopy Investigations

The transmission Mössbauer investigations based on ^57^Fe were carried out in order to identify multiple phases present in the as-prepared and annealed Fe-Co nanochains. Although this method is limited to iron-containing phases, if their content in the samples is substantial—as is the case of the tested samples, it can provide valuable information about their phase composition as well as hyperfine parameters.

**Figure 4:**
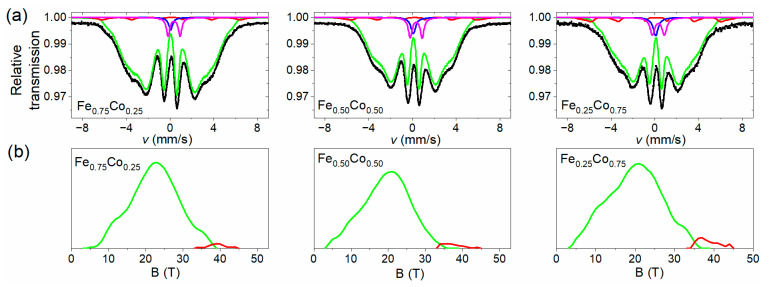
(**a**) Room temperature Mössbauer spectra collected for non-annealed Fe-Co nanochains, and (**b**) their corresponding hyperfine magnetic field distributions; component 1—green line, component 2—red line, component 3—violet and blue lines.

The room temperature Mössbauer spectra collected for as-prepared Fe-Co nanochains are shown in Figure 4a. The spectra have a smeared shape, characteristic of strongly disordered systems. A component characterized by a wide hyperfine magnetic field (HMF) distribution is the dominant part of each spectrum and any sharp sextets are not detected. There are small non-magnetic components in the central part of the spectra. In addition, the shape of Mössbauer spectra was reproduced by means of two Zeeman sextets with hyperfine field distribution (component 1 and 2 corresponding to green and red line in Figure 4a, respectively) combined with a centrally located non-magnetic subspectrum comprising a singlet and doublet (component 3 corresponding to violet and blue lines in Figure 4a). These distributions are presented in Figure 4b. Analyzing them, one can see that they have a smooth shape. The larger part of distribution comprises low and medium values of hyperfine magnetic field (up to 38 T) and lower isomer shift (*IS* = 0.12–0.17 mm/s), whereas the smaller one covers mainly higher values of HMF (35–50 T) and higher isomer shift (*IS* = 0.23–0.30 mm/s). Both the components are characterized by values of quadrupole splitting close to zero. The ranges of both distributions were chosen on the basis of the quality of the spectral fit. Relative intensities of the components (*p*) and their mean HMF field values (<B>) are collected in Table 2.

Undoubtedly, the individual parts of HMF distribution have different origins. The larger part related with component 1 represents atoms situated in the cores of spherical particles composing Fe-Co nanochains, in the form of an amorphous alloy containing mainly iron and cobalt. The amorphous state of the cores is stabilized by remnants of the manufacturing procedure, including boron, carbon, possibly oxygen, and other elements identified in investigations performed by means of EDS technique. The high-field part of HMF distribution associated with component 2 is attributed mainly to the oxide shell composed of iron and/or iron-cobalt oxides in a form of crystallites that are strongly distorted, primarily due to the proximity of both surfaces (inner and outer) of the oxide layer. However, the mean HMF values of this component are clearly smaller than typical of most crystalline Fe and Fe-Co oxides [31,32,33,34]. This can indicate the fine-grained character of these crystallites. Moreover, taking into account the fact that the separation of both HMF distributions is ambiguous (especially in the area of overlapping field ranges), it cannot be ruled out that a small amount of crystalline Fe-Co phase is also present in the samples which makes contribution to the HMF distribution in the field range about 36–39 T and, thus, further reduces the mean value of the HMF. Amorphous iron oxides are non-magnetic at room temperature [35,36] and, if they are present in a small amount in the nanochains, they contribute to the central doublets.

The central part (component 3) covers 5–8% of the spectrum and is composed of a doublet with a line width of about 0.4 mm/s, quadrupole splitting 1.0–1.1 mm/s and isomer shift 0.45–0.48 mm/s (approx. 2/3 of the central component intensity), and a singlet with similar line width and isomer shift 0.17–0.19 mm/s. They are attributed to fine particles of iron-cobalt (a singlet) or iron and iron-cobalt oxides (a doublet), which show superparamagnetic behavior at room temperature, including also oxides in the amorphous state.

**Figure 5:**
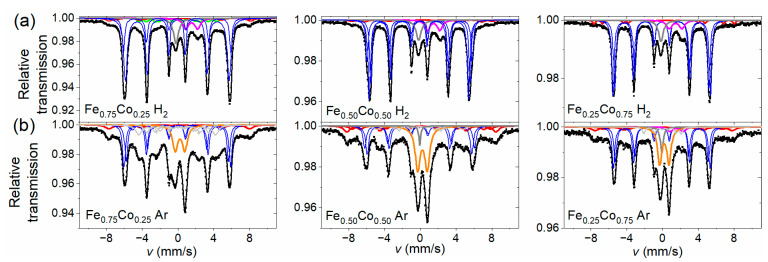
Room temperature Mössbauer spectra collected for the Fe-Co nanochains annealed in (**a**) hydrogen-containing atmosphere, and (**b**) argon atmosphere; component I—blue lines, component II—red line, component III—orange line, and component IV—olive and grey lines.

The Mössbauer spectra of the thermally treated Fe-Co nanochains shown in Figure 5 exhibit quite different shape in comparison with those obtained for their non-annealed counterparts. They consist of a number of rather sharp components characteristic for crystalline phases. Some their parameters determined using the fitting procedure are presented in Table 3. In the case of nanochains annealed in hydrogen-containing atmosphere (cf. Figure 5a), the dominant part of each spectrum (component I), of total relative intensity 74–76%, is composed of three or two Zeeman sextets with isomer shift close to zero and HMF values between 32–38 T. This component is attributed to the crystalline Fe-Co phase forming cores of nanochains and possibly containing a trace amount of residues from the manufacturing procedure. The HMF values derived for the sextets decrease with increasing cobalt content, which is in line with the observations reported for bulk [37] and nano-sized [38] iron-cobalt alloys as well as the iron-cobalt nanowires [39]. Spectrum obtained for the sample Fe_0.75_Co_0.25_ H_2_ also comprises a small (6%) component IV in a form of a set of more smeared sextets corresponding to the HMF values of about 16, 24, and 28 T. They might be attributed to (Fe-Co)_3_C or other Fe-Co-B-C structures [40,41]. However, it is more likely that these sextets represent different chemical surroundings in the two first coordination zones in the disordered Fe-Co alloy [42].

In turn, the high-field part of the spectra recorded for samples annealed in hydrogen-containing atmosphere was reproduced by two Zeeman sextets with isomer shift between 0.2 and 0.4 mm/s as well as the HMF values of about 41 and 49 T (component II). Its percentage is 4–7% and it represents the shell of distorted iron-cobalt oxides (mainly CoFe_2_O_4_), which covers the core of nanoparticles comprising the nanochains. Relative intensity of this component is very similar to that of the corresponding non-annealed samples except for the sample containing nominally 25% of iron where the intensity of this component is about half lower. This means that heating in the hydrogen-containing atmosphere did not increase or even slightly reduced the relative share of the oxide layer.

In addition, a singlet with isomer shift of about −0.08 mm/s (7–10%) and a wide doublet with isomer shift 1.6 mm/s and quadrupole splitting 1.4 mm/s were found in the central part of the spectra. The former one can be related to the superparamagnetic, small iron groupings located in the fcc cobalt lattice [42]. The latter component comes from an unidentified phase containing Fe^2+^. The relative share of the whole non-magnetic component ranges from 17% to 19% (component III).

In the spectra collected for the Fe-Co nanochains annealed in argon atmosphere (shown in Figure 5b), similar components are present, but proportions between them are different than in the previous series. Zeeman sextets representing iron or iron-cobalt oxides make 15–25% of the total spectrum (component II). This means that the oxide shell of nanoparticles is larger in these nanochains. In addition, the presence of a sextet with isomer shift of 0.31 mm/s, quadrupole splitting −0.05 mm/s, and HMF equal to 51.7 T was found, which is attributed to FeCo_2_O_4_ phase [32]. The sub-spectrum corresponding to iron-cobalt phases (component I) covers 57%, 38% and 43% for samples with nominal iron percentages of 75%, 50%, and 25%, respectively, therefore, it is clearly smaller than in the series of nanochains heated in hydrogen-containing atmosphere. Instead, the central part of the spectrum increased, comprising a singlet (component IV) and a doublet (component III) of isomer shift 0.30–0.35 mm/s and quadrupole splitting 1.0–1.1 mm/s with relative intensity between 17% and 33%. This doublet is attributed to the fine, superparamagnetic CoFe_2_O_4_ particles. Because the critical size of such particles at room temperature was about 10 nm [33,43], it can be concluded that some of them are smaller in size than this value.

### 3.5. Magnetic Investigations

**Figure 6:**
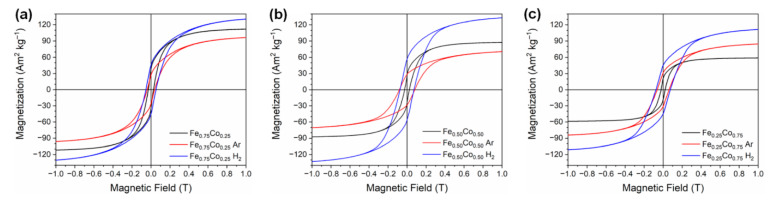
Room temperature magnetic hysteresis loops of as-prepared and annealed (**a**) Fe_0.75_Co_0.25,_ (**b**) Fe_0.50_Co_0.50_, and (**c**) Fe_0.25_Co_0.75_ Wire-like nanochains.

The results of room temperature magnetic analyses performed for all investigated Fe-Co nanostructures are demonstrated in Figure 6 and summarized in Table 4. Analyzing the presented data, one can see that the as-prepared as well as annealed Fe-Co nanochains reveal the ferromagnetic response with respect to the applied magnetic field. It is also observed that the coercivities (*H*_C_) of all annealed Fe-Co nanochains are higher in comparison with their non-annealed counterparts. Nevertheless, their values are comparable for both annealing atmospheres. Furthermore, the saturation magnetization (*M*_S_) of the Fe_0.25_Co_0.75_ nanochains annealed in both atmospheres is higher than that for the non-annealed sample. In turn, the *M*_S_ of Fe_0.75_Co_0.25_ and Fe_0.50_Co_0.50_ chains annealed in argon are lower than those recorded for non-annealed samples.

## 4. Discussion

As stated in the Introduction section, the nanomaterials synthesized with NaBH_4_ usually reveal amorphous or nanocrystalline nature. In fact, this is confirmed in this work. According to the XRD results, the as-prepared Fe_1−*x*_Co*_x_* (*x* = 0.25; 0.50; 0.75) Wire-like nanochains synthetized in the MFI co-precipitation reaction reveal only two very broad peaks whose positions are hardly difficult to assign the well-defined crystallographic planes of bcc Fe-Co phase. Moreover, the Mössbauer spectra reveal a smeared shape that is characteristic for strongly disordered systems. Therefore, the post-synthesis annealing at 400 °C in two relatively inert atmospheres, i.e., a mixture of 80% nitrogen and 20% hydrogen, as well as >99% argon gas, was applied in order to improve their crystallinity as well as to investigate the evolution of their structural and magnetic properties.

The experimental results obtained in this work indicate that the nominal structural and magnetic properties of the annealed Fe-Co nanochains depend on two factors. The first one is the chemical composition and structure of as-prepared material, whereas the second is related to the applied atmosphere during the thermal treatment. According to the electron microscopy data, the as-prepared Fe-Co nanochains are very similar in terms of their sizes as well as external and internal structures. They are composed of nanoparticles aligned in the nearly straight chains. Moreover, they exhibit characteristic core-shell structures, in which the thick core is covered by the thin shell layer reaching only about 4 nm. Undoubtedly, the presence of a shell layer is associated with an initial oxidation of investigated materials, which was confirmed by the performed EDS and TMS measurements. Besides that, this phenomenon has been frequently observed for the nanomaterials composed of iron and/or cobalt, which stay in contact with atmospheric air [9,19,20,21,25,38,42]. The thickness of initial oxide shells measured for the nanochains investigated in this work is very similar to those already reported in literature [9,17,24,26]. Apart from the oxide shell formation, the EDS results indicate that as-prepared Fe-Co nanochains are partially contaminated by the compounds containing boron and sodium (cf. Table 1). Their origin might be associated with either the unreacted NaBH_4_ taken to the synthesis or the by-products of the process, for instance, B(OH)_3_. Also, it cannot be excluded that some of the boron forms the borides with the Fe, Co, or both these elements. On one hand, the EDS technique is not sufficient for the determination of the level of light elements (e.g., boron), in particular, in small quantities. On the other hand, it is well known that the presence of boron often causes the amorphization of various inorganic compounds [18]. This is associated with the fact that boron reveals extremely complex chemistry because it can form various metal borides with very different stoichiometry, which are not compatible with standard concepts of chemical bonding. Taking into account the specific features of boron mentioned above, it is believed that this element might be present in the as-prepared Fe-Co nanochains causing their amorphization. This, in turn, encounters the problems with interpretation of the XRD, TMS, and magnetic results obtained for those samples. It should be also mentioned that the formation of boride has been already reported for the Co nanoparticles [44,45], the Fe nanoparticles [46,47], as well as the Fe-Co nanoparticles [26,48].

The EDS results measured for the as-prepared Fe-Co nanochains indicate that the purification process leads to the complete removal of chloride ions from the samples. They also confirm that the actual share between Fe and Co atoms in the as-prepared samples corresponds well to the composition of initial ion precursors taken to the MFI synthesis.

Analyzing the electron microscopy images recorded for the thermally-treated Fe-Co nanochains, one can see that their annealing performed in hydrogen-containing atmosphere does not influence their size. In turn, the process in argon causes (i) an increase of their diameters, (ii) a growth of shell layer, as well as leads to the roughening of their surfaces. Undoubtedly, these observations are related to a partial oxidation of the Fe-Co nanochains. This finding is also confirmed by the XRD, TMS, and magnetic measurements. Considering the XRD and TMS results, it is clear that the ratios of the relative intensities between oxide and alloy phases are much higher for the annealing in argon. Moreover, the presence of oxides is hardly noticeable in the XRD patterns recorded for the Fe-Co nanochains annealed in hydrogen-containing atmosphere. Therefore, it is assumed that a very small content of oxide in these samples might be associated with either crystallization of internal oxides or surface oxidation caused by their storage in atmospheric air. In turn, the magnetic tests indicate that the *M*_S_ of the Fe_0.75_Co_0.25_ and Fe_0.50_Co_0.50_ nanochains annealed in argon are lower, whereas their *M*_S_ in hydrogen-containing atmosphere are higher than those recorded for non-annealed samples. This observation is associated with the partial oxidation during thermal processing in argon. At this point, it should be underlined that the Fe_0.25_Co_0.75_ sample thermally-treated in both annealing atmospheres reveals the presence of hcp Co phase, which is much more intense than the signal coming from oxides. Therefore, this additional phase hinders the oxidation of the Fe_0.25_Co_0.75_ nanochains. This reflects in their morphology and magnetic properties. Namely, the Fe_0.25_Co_0.75_ nanochains are covered by a nanosheet-like shell layer in the case of annealing performed in argon. Also, the *M*_S_ values recorded in hydrogen-containing atmosphere as well as in argon are higher than that for non-annealed sample due to the presence of hcp Co phase. It is also interesting that the *H*_C_ of all thermally-treated Fe-Co nanochains are higher in comparison with their non-annealed counterparts. The lowest increase of *H*_C_ is found for the annealed sample in which the content of cobalt is the lowest. In turn, the highest *H*_C_ value is recorded for the Fe_0.50_Co_0.50_ nanochains. These observations are not entirely clear because the *H*_C_ usually depends on many factors such as: shape, size, presence of internal impurities and/or stresses, ordering, and distances between neighboring chains [49], etc. Therefore, they are based on the obtained magnetization hysteresis curves rather than the physical explanations.

Reassuming, the above discussion of experimental results indicates that the annealing atmosphere is quite an important parameter in the case of amorphous Fe-Co Wire-like nanochains and can affect the final chemical and physical properties of thermally treated nanostructures.

## 5. Conclusions

This work presents how the annealing atmosphere influences the structural and magnetic properties of the amorphous Fe_1−*x*_Co*_x_* (*x* = 0.25; 0.50; 0.75) Wire-like nanochains, which were prepared in the magnetic-field-induced synthesis. In general, the two atmospheres were investigated, namely: (i) a mixture of 80% nitrogen and 20% hydrogen, and (ii) argon. Both of them are often recognized as inert atmospheres. Therefore, similar results were expected before performance of the experiments. However, the annealing in argon and hydrogen-containing atmospheres led to significantly different changes of structural and magnetic properties of the as-prepared Fe-Co samples.

The thermal processing in argon caused crystallization of the cores of the as-prepared Fe-Co nanochains in the form of bcc Fe-Co phase. At the same time, their diameters of the annealed samples became wider and their surfaces rougher due to the formation of a mixture of CoFe_2_O_4_ and Fe_3_O_4_ oxides. Moreover, it was found that the thermally-treated Fe_0.25_Co_0.75_ nanochains were covered by the nanosheet-like oxide shell, which constituted a better barrier against oxidation than oxide layers observed in the case of the annealed Fe_0.75_Co_0.25_ and Fe_0.50_Co_0.50_ samples. The annealing performed in hydrogen-containing atmosphere also caused crystallization of the cores of the as-prepared Fe-Co nanochains in the form of bbc Fe-Co phase, but their initial oxide shell layers decompose. It is also worth adding that apart from the bcc Fe-Co phase, the cores of the Fe_0.25_Co_0.75_ nanochains thermally-treated in both argon and hydrogen-containing atmosphere included hcp Co phase. According to the magnetic measurements, the *H*_C_ of annealed Fe-Co nanochains increased in comparison with their non-annealed counterparts. It was also found that the *M*_S_ of the Fe_0.25_Co_0.75_ nanochains annealed in both atmospheres were higher than that for non-annealed sample, whereas the *M*_S_ of the Fe_0.75_Co_0.25_ and Fe_0.50_Co_0.50_ samples annealed in argon were lower than those recorded for non-annealed nanochains. This observation is related to their partial oxidation during thermal treatment. Finally, it is worth adding that the obtained results can be considered as an important source of information for the application of thermally-treated Fe-Co nanochains as magnetic and microwave absorbers [27,28] or in catalysis [29,30].

## Figures and Tables

**Table 1:** Weight and atomic percentage of elements forming the as-prepared Fe_0.75_Co_0.25_, Fe_0.50_Co_0.50_, and Fe_0.25_Co_0.75_ nanochains, and carbon type determined with EDS technique.

Element	Percentage	Fe_0.75_Co_0.25_	Fe_0.50_Co_0.50_	Fe_0.25_Co_0.75_	Carbon Tape
B	weight	2.1	3.3	1.2	0.30
	atomic	7.4	11.2	4.6	0.30
C	weight	5.2	2.4	1.0	94.60
	atomic	16.7	7.4	3.4	95.80
O	weight	6.5	10.5	10.4	5.10
	atomic	15.7	24.0	26.5	3.80
Na	weight	1.4	3.6	3.5	-
	atomic	2.4	5.7	6.3	-
Fe	weight	65.7	45.9	23.9	-
	atomic	45.4	30.0	17.6	-
Co	weight	19.0	33.7	59.9	-
	atomic	12.4	20.9	41.6	-
Fe	normalized weight ^1^	77.6	57.7	28.5	-
	normalized atomic ^1^	78.5	58.9	29.7	-
Co	normalized weight ^1^	22.4	42.3	71.5	-
	normalized atomic ^1^	21.5	41.1	70.3	-

^1^ The values of normalized weight and atomic percentage of Fe and Co elements were determined considering the EDS signal associated with them and avoiding the signal of other elements.

**Table 2:** Parameters of components of Mössbauer spectra collected for non-annealed Fe-Co nanochains: p—relative intensity, <B>—mean HMF. Components: component 1—low- and middle- field part of spectrum, component 2—high-field part of spectrum, and component 3—paramagnetic and/or superparamagnetic component.

	Component 1		Component 2		Component 3	Mean HMF
	p (%)	<B> (T)	p (%)	<B> (T)	p (%)	<B>_sample_ (T)
Fe_0.75_Co_0.25_	93	22.4	2	39.1	5	21.6
Fe_0.50_Co_0.50_	90	19.4	2	37.4	8	18.4
Fe_0.25_Co_0.75_	88	19.9	4	38.6	8	19.0

**Table 3:** Parameters of components of Mössbauer spectra collected for Fe-Co nanochains annealed in hydrogen-containing atmosphere and in argon: p—relative intensity, <B>—mean HMF. Components: component I—iron-cobalt alloy, component II—iron and/or iron-cobalt oxides, component III—paramagnetic and/or superparamagnetic component, and component IV—other magnetic phases mentioned in text.

	Component I	Component II	Component III	Component IV	Mean HMF	Mean HMF
	p (%)	p (%)	p (%)	p (%)	<B>_Fe-Co_ (T)	<B>_sample_ (T)
Fe_0.75_Co_0.25_ H_2_	74	6	14	6	36	30
Fe_0.50_Co_0.50_ H_2_	75	7	18	0	35	29
Fe_0.25_Co_0.75_ H_2_	76	4	20	0	33	27
Fe_0.75_Co_0.25_ Ar	57	15	17	11	35	28
Fe_0.50_Co_0.50_ Ar	38	25	33	4	36	27
Fe_0.25_Co_0.75_ Ar	43	22	31	4	33	25

**Table 4:** Coercivity (*H*_C_) and saturation magnetization (*M*_S_) of as-prepared and annealed Fe-Co nanochains determined during the room temperature magnetic measurements.

Sample	*H*_C_ (Oe)	*M*_S_ (Am^2^·kg^−1^)
Fe_0.75_Co_0.25_	273	112
Fe_0.75_Co_0.25_ H_2_	528	139
Fe_0.75_Co_0.25_ Ar	416	109
Fe_0.50_Co_0.50_	246	88
Fe_0.50_Co_0.50_ H_2_	735	141
Fe_0.50_Co_0.50_ Ar	808	80
Fe_0.25_Co_0.75_	215	59
Fe_0.25_Co_0.75_ H_2_	757	118
Fe_0.25_Co_0.75_ Ar	651	94

## Data Availability

The data presented in this study are available on request from the corresponding author.
